# Ion channel diversity, channel expression and function in the choroid plexuses

**DOI:** 10.1186/1743-8454-4-8

**Published:** 2007-09-20

**Authors:** Ian D Millar, Jason IE Bruce, Peter D Brown

**Affiliations:** 1 Faculty of Life Sciences, Core Technology Facility, University of Manchester, Manchester M13 9NT, UK

## Abstract

Knowledge of the diversity of ion channel form and function has increased enormously over the last 25 years. The initial impetus in channel discovery came with the introduction of the patch clamp method in 1981. Functional data from patch clamp experiments have subsequently been augmented by molecular studies which have determined channel structures. Thus the introduction of patch clamp methods to study ion channel expression in the choroid plexus represents an important step forward in our knowledge understanding of the process of CSF secretion.

Two K^+ ^conductances have been identified in the choroid plexus: Kv1 channel subunits mediate outward currents at depolarising potentials; Kir 7.1 carries an inward-rectifying conductance at hyperpolarising potentials. Both K^+ ^channels are localised at the apical membrane where they may contribute to maintenance of the membrane potential while allowing the recycling of K^+ ^pumped in by Na^+^-K^+ ^ATPase. Two anion conductances have been identified in choroid plexus. Both have significant HCO_3_^- ^permeability, and may play a role in CSF secretion. One conductance exhibits inward-rectification and is regulated by cyclic AMP. The other is carried by an outward-rectifying channel, which is activated by increases in cell volume. The molecular identity of the anion channels is not known, nor is it clear whether they are expressed in the apical or basolateral membrane. Recent molecular evidence indicates that choroid plexus also expresses the non-selective cation channels such as transient receptor potential channels (TRPV4 and TRPM3) and purinoceptor type 2 (P2X) receptor operated channels. In conclusion, good progress has been made in identifying the channels expressed in the choroid plexus, but determining the precise roles of these channels in CSF secretion remains a challenge for the future.

## 1. Introduction: 25 years of the patch clamp method

It is now more than 25 years since the publication of the seminal paper which first described the patch clamp method for studying ion channels [1]. In recognition for their work in developing the patch clamp method Bert Sackmann and Erwin Neher, two of the authors on this original paper, were awarded the 1991 Nobel Prize for Medicine. The impact of the method is perhaps most obvious in studies of the activity of individual ion channels (single channel recording). This configuration of the method gives scientists the unique opportunity to study the activity of a single protein. Used in conjunction with recombinant DNA techniques, this method has vastly increased our understanding of how protein structure relates to channel function. In recognition of such studies the 2003 Nobel Prize for Chemistry was awarded to Rod McKinnon (jointly with Peter Agre).

Patch clamp methods, particularly whole cell methods, have also been important in determining the physiological roles of channels in mammalian cells. This is particularly true in secretory epithelia, where scientists such as Ole Petersen and Alain Marty pioneered the use of whole cell methods to study the mechanisms of secretion in exocrine acinar cells [2,3]. In fact the one major refinement of the technique, the "perforated patch", was developed by Marty and Horn while working on lacrimal gland acinar cells [4]. The immense impact of patch clamp methods to disciplines such as physiology and neuroscience is perhaps best illustrated by the fact that a total of 100 000 papers which used the patch clamp method were published between 1981 and 2001 [5].

Patch clamp methods were originally applied to choroid plexus over 20 years ago; first in amphibian tissue and subsequently in mammalian tissue. The impact of the technique to understanding choroid plexus physiology, however, is less dramatic than that in studies of secretory, exocrine acinar cells. There are probably for two main reasons for this: 1) choroid plexus cells are less robust than exocrine acinar cells, thus it has been more difficult to perform complex experiments, 2) exocrine acinar cells express relatively few channels, while in comparison the choroid plexus cells express a wide range of ion channels making the separation of distinct transport pathways more problematic. In recent years, however, the molecular structures of most ion channels have been determined. As a result molecular localisation techniques such as *in situ *hybridisation, reverse transcriptase polymerase chain reaction (RT-PCR), Western blotting and immunocytochemistry have been employed to resolve many of the complexities of channel expression in the choroid plexus. This article will discuss these data. It will also speculate on future areas of development. To provide a frame work for this discussion we first give a brief general overview of ion channel structure and function.

The reader is reminded that while there is little doubt that ion channels have many important roles in the choroid plexus, many other transport proteins (pumps and carriers) are expressed in the choroid plexus. These are not discussed in this article but are the subject of two recent reviews [6,7].

## 2 Ion channel diversity

Ion channels are expressed in all cells. They are integral membrane proteins that form selective pores in cell membranes (often as multimers), which facilitate the movement of ions across the membrane down their electrochemical gradient. They are characterised by high rates of transport (millions of ions.s^-1^) compared to other transport proteins e.g. facilitated glucose (GLUT) transporters which transport about 100 molecules.s^-1^. This high rate of transport is important because it means that ion movements can create significant changes in the electrical properties of a cell, it also means that ion channels are exploited as a point of regulation in most cells. Thus many different mechanisms have evolved by which channel activity can be modulated, e.g. voltage, ligand binding, phosphorylation and mechanical stress.

There is no simple, systematic nomenclature for ion channels. However, in general they are classified primarily by reference to the ion to which they are selective, i.e. K^+^, Na^+^, Ca^2+^, anions. They are then sub-divided on the basis of functional properties such as a mechanism of regulation (e.g. Ca^2+^-activated) or a biophysical characteristic (e.g. inward rectifier). Even with increased knowledge of channel molecular structure this simple classification based on selectivity still works well. However, there are two classes of channel that do not adhere to these simple rules: the receptor-operated channels (selective for either cations or anions) and novel transient receptor potential (TRP) channels (which discriminate poorly between monovalent and divalent cations). Each broad group of channels has many members, and can be further sub-divided as will be seen below.

### 2.1 Potassium channels

**Figure 1:**
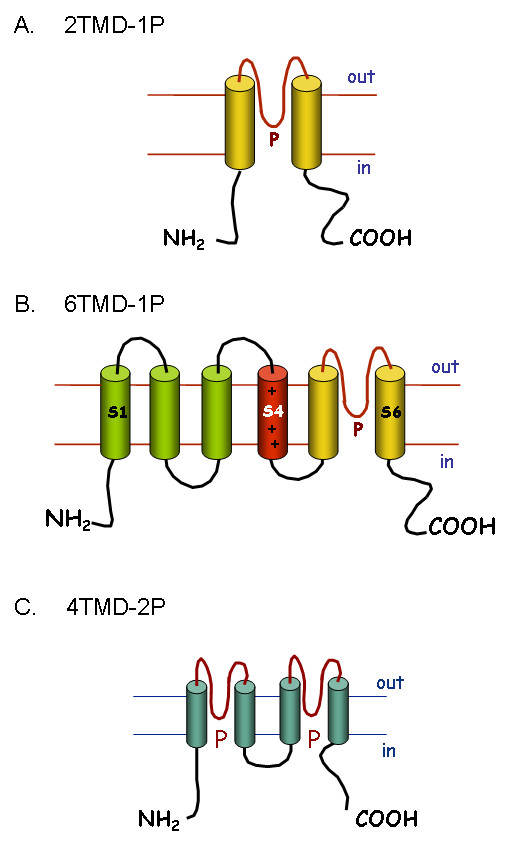
**K^+ ^channel structures**. Secondary structures of pore forming α-subunits of the: A) 2TMD-1P Kir channels, B) 6TMD-1P voltage-gated K^+ ^channels and C) 4TMD-2P twin pore domain K^+ ^channels. Functional channels are a tetramer of α-subunits (or dimer in the case of the 4TMD-2P), arranged so the extracellular P loops form the pore region of the channel. The charged TMD S4 in the 6TMD-1P structure is thought to be involved in the voltage-gating of these channels. Channel gating may also be modified by the presence of auxiliary β-subunits.

The potassium channels are the largest and most complex family of ion channels, represented by at least 70 loci in the human genome [8]. All K^+ ^pore-forming channel proteins (α-subunits) share a conserved region of two transmembrane domains (TMD) which are linked by an extracellular or pore (P) domain (Figure 1). This P domain is thought to form the pore region of the channel, in what is generally a tetrameric channel complex. K^+ ^channels can be divided into three major families based on protein structure. The inward-rectifying channels (Kir) are structurally the simplest, with each subunit composed of the basic 2 TMD and a single P domain (2TMD-1P; see Figure 1A). A second large group of channels have an additional four TMDs adjacent to the 2TMD-1P structure these are the voltage-gated K^+ ^or 6TMD-1P channels (Figure 1B). The most recently identified family are twin-pore domain K^+ ^channels (4TMD-2P), which show a repeat of the 2TMD-1P structure (Figure 1C).

#### 2.1.1 Inward-rectifying K^+ ^channels (2TMD-1P)

These channels are known as inward-rectifying channels since they allow the passage of more inward K^+ ^current into the cell than outward K^+ ^current. They are widely expressed in many different types of cell throughout the body. The molecular identities of Kir1.1 (ROMK; from kidney distal convoluted tubule) and Kir2.1 (from a macrophage cell line) were first determined in 1993 by expression cloning [9,10]. Related channels in a total of seven sub-families (Kir1 to Kir7) have been subsequently cloned by homology methods [11]. Functional channels are formed by four α subunits. Each sub-family exhibits slight functional differences such as the degree of rectification and the mechanism of channel regulation. Two particularly important subfamilies are the Kir 3 proteins which include the G-protein regulated channels (GIRKs) found in cardiac muscle, and Kir6.1 and Kir6.2 which combine with the sulphonylurea receptor proteins to produce functional ATP-sensitive K^+ ^channels [11].

#### 2.1.2 Voltage-gated K^+ ^channels (6TMD-1P)

This is a large and diverse sub-family of K^+ ^channels with some 40 members [8]. The first member of the family to be identified was the *Shaker *channel in *Drosophila *[12]. Homology methods subsequently identified other K^+ ^channels in *Drosophila *and mammalian species. Most of the 6TMD-1P channels exhibit voltage-dependent gating, by virtue of the charged amino acids in the fourth TMD or S4 (Figure 1B). Functional channels are composed of a tetramer of α-subunits. Some β-subunits have also been identified which modify channel gating. The majority of the 6TMD-1P channels are classed as delayed-rectifier (Kv) channels, of which there are twelve families, i.e. Kv1 to Kv12 [8].

Kv1 to Kv4 represent the classical delayed-rectifier channels which are widely distributed in many cell types around the body. These channels exhibit voltage-dependent kinetics, opening when the membrane potential (Vm) is depolarised. The rate at which the channel opens on depolarisation in neurons, however, is slower than that for the activation of voltage-gated Na^+ ^channels, hence the name delayed-rectifying channels. The pronounced outward-rectification of the current-voltage relationships for these channels is largely a product of the voltage-dependent opening of the channel. Kv5, 6, 8 and 9 are structurally related to the delayed-rectifiers, however, they do not act as channels but are classed as modifiers [8]. Kv7 includes five proteins, previously known as the KCNQ channels, which have roles such as damping neuronal activity. Kv10, Kv11 and Kv12 show less structural homology to the classic Kv channels and originally were classified as the *eag, erg *and *elk *channels respectively [8].

A second major group in the 6TMD-1P family are the Ca^2+^-activated K^+ ^channels: "maxi" Ca^2+^-activated K^+ ^channels (BK_Ca_), intermediate conductance Ca^2+^-activated K^+ ^channels (IK_Ca_) and small conductance Ca^2+^-activated K^+ ^channels (SK_Ca_) [13]. These channels are all activated by increases in intracellular Ca^2+^. In the case of BK_Ca _this involves Ca^2+ ^binding to the channel protein, whereas Ca^2+ ^acts via calmodulin to open SK_Ca _and IK_Ca _[13]. BK_Ca _is also activated by depolarising potentials because of the voltage-dependent binding of Ca^2+ ^to the channel protein, but neither SK_Ca _nor IK_Ca _exhibit any voltage-dependence. The Ca^2+^-activated channels can be distinguished from one another by the size of the single channel conductance and by different sensitivities to a range of peptide toxins [13].

#### 2.1.3 Twin pore domain K^+ ^channels (4TMD-2P)

This family of 15 channels was identified just over a decade ago [14,15]. Each channel subunit has four TMDs and two P domains, i.e. they resemble two 2TMD-1P proteins linked together (Figure 1C). Dimers of these subunits form functional channels that are K^+ ^selective. They were first described as "background" or "leak" K^+ ^channels important in setting the resting Vm [16]. More recent studies suggest that they are regulated by a wide range of factors, e.g. pH, volatile anaesthetics and mechanical stress. They may therefore have specific roles in controlling Vm and cellular activity in many different types of cell [15,16].

### 2.2 Sodium channels

#### 2.2.1 Voltage-gated Na^+ ^channels

**Figure 2:**
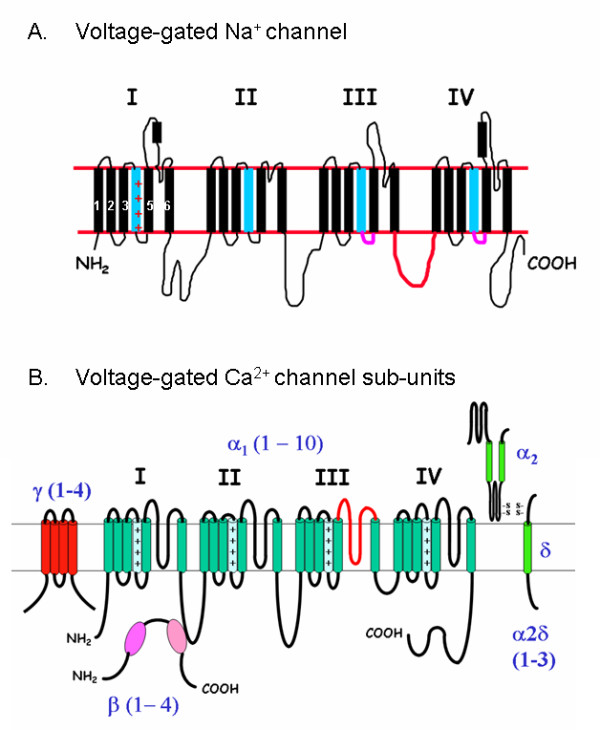
**Structures of A) voltage-gated sodium and B) voltage-gated Ca^2+ ^channels**. A) Secondary structure of the α-subunit of a voltage-gated Na^+ ^channel showing four homologous domains, each of six transmembrane spanning segments (S1–S6). Pore loops link the S5 and S6, the positively charged S4 (blue) acts the voltage sensor and a short intracellular loop (red) linking domains III and IV acts as the inactivation gate. In a functional channel the domains are arranged so that the pore loops from each domain form the pore of the channel. Functional channels also require one or two modulatory β-subunits for activity. B) The α-sub-units of voltage-gated Ca^2+ ^channels have a similar structure to that of the Na^+ ^channels, and are the pore forming subunits of the channel. Functional channels however have a more complex structure an require the presence of β, γ and δ subunits.

Voltage-gated Na^+ ^(Na_v_) channels are activated and then display rapid inactivation at depolarising Vm. The activity of Na_v _channels is vital in the generation of action potentials in nerve cells. They are composed of a pore-forming α-subunit which is usually associated with two modulatory β-subunits, except in skeletal muscle where only one β-subunit is required [17]. The α-subunit is composed of more than 1800 amino acids, which are divided into 4 homologous domains, each comprised of six transmembrane spanning segments (S1–S6; Figure 2A). A pore loop links S5 and S6, whilst positive charged amino acids are abundant in S4 which acts the voltage sensor. The domains are arranged so that the pore loops from each domain form the pore of the channel. A short intracellular loop that links domains III and IV acts as the inactivation gate (red in Figure 2A). Nine different α-subunits have been identified and together they form the Na_v _gene family [17]. The Na_v _proteins show tissue specific distribution, but they are expressed only in excitable cells (e.g. nerve and muscle) and cells which exhibit action-like potentials (e.g. pancreatic β-cells) [17].

#### 2.2.2 Epithelial Na^+ ^channels

Epithelial Na^+ ^channels(ENaC) were first described in frog skin, but are now known to be expressed in many mammalian epithelia involved in Na^+ ^absorption, e.g. distal convoluted tubule of the kidney, colon, lung and salivary gland duct [18]. By contrast to the Na_v _channels, ENaC are not gated by voltage and are characteristically blocked by low concentrations (<10 μM) of the diuretic drug amiloride. Three homologous subunits have been identified (α, β and γ). Each subunit is comprised of two TMD and a complex extracellular loop [18]. Functional channels are thought to be heterotetramers of two α, one β and one γ subunits. This protein structure reveals that ENaC is a member of the degenerin (DEG) family of channels. This name originates from studies of nematodes where the expression of mutant DEG channels leads to cellular degeneration [18]. Other members of the DEG family in mammals include acid-sensitive channels (ASIC) which may be involved in pain transduction [18].

### 2.3 Calcium channels

#### 2.3.1 Voltage-gated Ca^+ ^channels

Voltage-gated Ca^2+ ^channels (Ca_v_) are activated by Vm depolarisation and mediate Ca^2+ ^influx into so called "excitable" cells. Functional channels are composed of four or five subunits: α_1_, α_2_, β, δ and in some cells γ (Figure 2B). The α_1 _subunit is the largest subunit and determines most of the functional properties of the channel: pore structure, gating and pharmacology. The α_1_-subunits share a similar structure to those of the α-subunits of voltage gated Na^+ ^channels, i.e. they have four domains each composed of six transmembrane segments. Three families of α_1 _subunit have been identified [19]. The Ca_v_1 family contains the four L-type Ca^2+ ^channels which are expressed in muscle, neurons and endocrine cells. The neuronal specific P/Q, N and R type channels form the Ca_v_2 family. While the three T-type channels expressed in neurons and muscle comprise the Ca_v_3 family.

#### 2.3.2 Capacitative or Store-Operated Ca^2+ ^Entry

It was recognised in the early 1970's that agonist-evoked cytosolic Ca^2+ ^mobilisation in non-excitable cells involves a transient Ca^2+ ^release from intracellular stores, followed by a sustained Ca^2+ ^entry [20,21]. This led to the concept of "capacitative Ca^2+ ^entry" or store-operated Ca^2+ ^entry (SOCE), by which the depletion of intracellular stores leads to sustained Ca^2+ ^entry [22]. Electrophysiological studies have subsequently identified a Ca^2+ ^release activated current (I_CRAC_), which is characterised by inward rectification, very positive reversal potential (>30 mV), a high Ca^2+ ^selectivity (P_Ca_/P_Na_~1000), inhibition by La^3+ ^and low single channel conductance (<100 femto siemens, fS) [23]. The molecular identity of this channel and the mechanism that couples store depletion to Ca^2+ ^entry, however, have remained largely unknown until very recently.

The last two years have seen major advances in our understanding of SOCE, with the discovery of two important proteins, stromal interation molecule (STIM1) and CRAC modulator (CRACM or Orai1) [24-28]. STIM1 contains a Ca^2+^-binding domain (EF hand) that has been suggested to sense endoplasmic reticulum Ca^2+ ^store depletion [24,25]. Whereas Orai1, a four transmembrane domain protein, is suggested to be the pore forming subunit of the CRAC channel [27]. Interference RNA knockdown (siRNA) of either STIM1 or Orai1 significantly reduces SOCE and I_CRAC _[24,26], whereas co-expression of both STIM1 and Orai1 massively increases SOCE and I_CRAC _[29,30]. Mutagenesis of residues within Orai1, predicted to be important for Ca^2+ ^binding within the pore of the channel, also markedly attenuated SOCE and I_CRAC _[27]. Collectively these data suggest that Orai1 acts as a Ca^2+ ^entry channel [31,32].

### 2.4 Anion channels

Research performed over the last two decades has illuminated the importance of anion channels in many physiological and patho-physiolgical processes [33]. Knowledge of the molecular physiology of anion channels, however, is very limited compared to that of cation channels. Definitive information is available on the structure and function of only two classes of channel (e.g. the cystic fibrosis transmembrane conductance regulator and the voltage dependent Cl^- ^channels of the ClC family). The molecular identity of many other channels is either controversial (e.g. Ca^2+^-activated Cl^- ^channels) or unknown (e.g. volume-sensitive anion channels).

#### 2.4.1 Cystic fibrosis transmembrane conductance regulator

**Figure 3:**
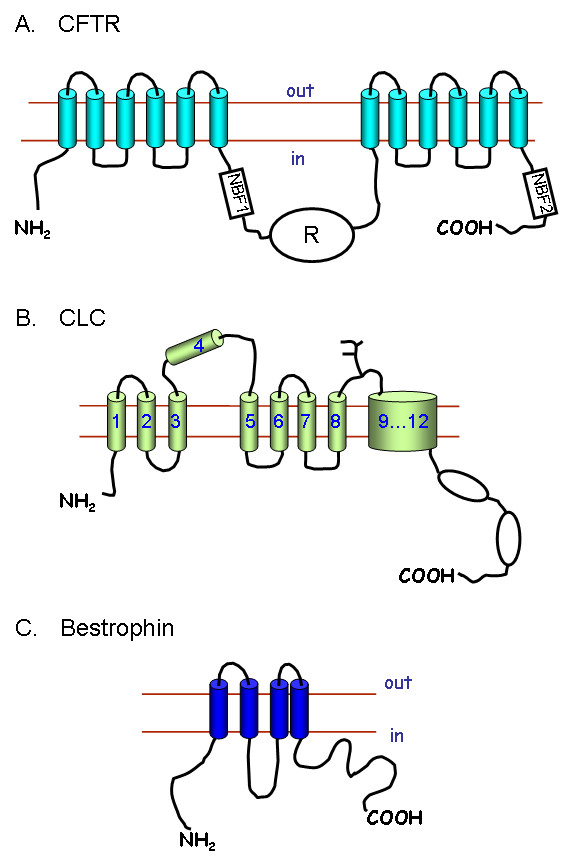
Anion channel structures. **A) **The cystic fibrosis transmembrane conductance regulator CFTR is composed of two repeats of six TMD linked by a regulatory subunit (R) and a nucleotide binding fold (NBF1). A second nucleotide binding fold (NBF2) is found towards the COOH-terminus of the protein. The predicted secondary structures of B) the voltage-gated ClC chloride channels and **C) **the bestrophin family are still the subject of some controversy, e.g. the precise number of transmembrane domains in the 9...12 region of the ClC channels is not known.

The cystic fibrosis transmembrane conductance regulator (CFTR) is the channel that is defective in the disease cystic fibrosis. The mRNA for the channel was identified by positional cloning, and encodes a protein of 1280 amino acids (Figure 3A). The structure of the protein is unique for an ion channel, and CFTR is in fact an ATP binding cassette (ABC) protein [34]. This unusual structure meant that when first cloned the protein was described as conductance regulator, and it was only after extensive series of experiments that channel function was established [35]. The CFTR channel has a single channel conductance of about 10 pico siemens (pS), it is primarily selective to Cl^-^, but also has a finite permeability to HCO_3_^- ^[36]. Channel activity is increased by phosphorylation with protein kinase A of serine residues in the regulatory domain (R; Figure 3A) of the protein. Channel activity is also dependent on ATP binding to the nucleotide binding folds (NBF1 and NBF2; Figure 3A). Over a 1000 mutations have been identified in patients suffering from cystic fibrosis. These mutations can affect trafficking of CFTR to the membrane, channel regulation or single channel conductance [37].

CFTR is expressed in organ systems affected by cystic fibrosis, e.g. airways epithelia, the exocrine pancreas, the small intestine, the biliary tract and the male reproductive tract [37]. However, it is also expressed in other tissues which are not thought to be affected by cystic fibrosis and where the function of CFTR remains unclear, e.g. cardiac muscle [38] and the kidney [39].

#### 2.4.2 The CLC family of channels and transporters

The only family of anion channels which has been well characterised by both molecular and functional methods is the ClC family. The voltage-dependent channel ClC-0 was originally cloned by expression methods from the electric organ of the torpedo ray [33]. ClC-1 the mammalian skeletal muscle Cl^- ^channel was subsequently cloned by homology methods [33]. Eight other members of the ClC family have now been identified, these include three Cl^- ^channels: ClC-2 which is widely expressed, and ClC-Ka and ClC-Kb from kidney. The other ClC proteins (ClC-3 to ClC-7) were initially classed as channels, but they are now thought to mediate the exchange of Cl^- ^and H^+ ^[33,40,41].

The ClC channels are thought to have at least 11 transmembrane spanning domains (Figure 3B), although precise structure remains uncertain [33]. The ClC channels are thought to function as dimers with a functional pore structure in each subunit. Although all the ClC channels appear to have a similar structures, they are functionally quite different in terms of voltage-dependent gating e.g. ClC-2 activates at hyperpolarising potentials whereas ClC-Kb is activated at depolarising potentials [33].

#### 2.4.3 Ca^2+^-activated Cl^- ^channels

The Ca^2+^-activated Cl^- ^channels are a group of channels which have important functions in fluid secretion by some epithelial cells [2,3], stimulus contraction coupling in smooth muscle cells [42] and in olfaction [43]. The molecular identity of these channels has not been established. One group of candidate proteins is the ClCA family of channels [42]. These proteins act as anion channels when expressed in mammalian cells, but their properties are significantly different from those of Ca^2+^-activated Cl^- ^channels in native tissues [42]. A second family of proteins the bestrophins, may also act as Ca^2+^-activated Cl^- ^channels. The structure (Figure 3C) and function of the bestrophin channels, however, has not been fully established [44]. The first bestrophin to be identified was best1 (in total there are four proteins encoded by the human genome: best1 to best 4), and mutations to this channel is associated with macular degeneration in the retina [44]. A very recent paper has shown that best 1 is expressed in a number of secretory epithelial cells, and that the use of siRNA against best 1 reduces the Ca^2+^-activated Cl^- ^currents in these cells [45].

#### 2.4.4 Volume-sensitive anion channels (VSAC)

These channels appear to be ubiquitously expressed [33]. They make a major contribution to cell volume regulation, and may also play a critical role in the events of the cell cycle [46]. They display outward rectification and often time-dependent inactivation at extreme depolarising Vm. A number of molecular candidates have been suggested including: p-glycoprotein, CLC3 and putative Cl^- ^channel protein (pI_Cln_), but none has been proved to be VSAC [33]. Indeed pI_Cln _and p-glycoprotein are now described as regulators of VSAC [33], while ClC-3 is probably an ion exchanger in the membranes intracellular organelles [41].

### 2.5 Receptor operated channels

The receptor operated channels (ROCs) are a diverse group of channels which are activated by the binding of an agonist to a receptor site that is part of the channel protein. They have many important roles, principally at synapses of the central nervous system (CNS). By contrast to most other channels they have not been classified in terms of the permeating ion, but rather by the name of the activating agonist. They can be divided into three major groupings: the classical ROCs, the glutamate receptors and the P2X receptors.

#### 2.5.1 Classical ROCs

The classical ROCs were the first to be identified in terms of function and then by molecular structure [47]. They all have a similar subunit structure with four TMD, and five subunits are needed to form a functional channel. They are activated by acetylcholine (the nicotinic ACh receptor; nACh), serotonin (5HT_3_), γ-amino butyric acid (GABA_A _and GABA_C_) and glycine. nACh and 5HT_3 _are cation selective, whereas the GABA_A_, GABA_C _and glycine channels are permeable to anions. The pore structure of the glycine and GABA channels are only a few amino acids different to those of other ROCs, but this small variation in amino acid sequence confers the anion selectivity on these channels [48]. These channels are normally associated with synapses in the CNS, but there is evidence that ROCs are expressed in other cells. For instance nACh plays a key role in transmission at the neuromuscular junction, while GABA_A _is expressed in glial cells in the CNS, peripheral nerves [49] and other cells, e.g. glucagon secreting alpha-cells of the endocrine pancreas [50].

#### 2.5.2 Glutmate receptors

The ionotropic glutamate receptors are functionally similar to the classic ROCs. They have important roles at synapses of the CNS, and are activated by glutamate or aspartate. However, they have a different structure to the classic ROCs. Each subunit has only three TMD, and functional channels are heteromers in which four subunits assemble as a "dimer of dimers" [51]. The glutamate receptors can be divided into three sub-families on the basis of their activation by different selective agonists: kainate, N-methyl-D-aspartate (NMDA) and α-amino-3-hydroxy-5-methyl-4-isoxazole propionic acid (AMPA). The different sub-families also show slightly different kinetic properties and ion selectivity, e.g. the AMPA and kainate channels are selective for monovalent cations and display rapid activation, whereas the NMDA channels exhibit slower activation and are permeable to both Na^+ ^and Ca^2+^.

#### 2.5.3. Type 2 purinoceptors

Type 2 purinoceptors (P2X receptors) are receptors for the purine nucleotides. They can be functionally and structurally divided into two families: the P2Y family are G-protein coupled receptors which are activated by ATP, UTP, ADP and UDP; and the P2X receptors which are receptor-operated channels which are activated primarily by ATP [52]. A total of seven P2X receptor proteins have been identified [53]. Each P2X protein has 2 TMD and functional channels are composed of trimers of three identical subunits, or a combination two different subunits. All P2X receptor channels are permeable to small monovalent cations, and some are also permeable to Ca^2+ ^and anions. They are widely distributed throughout the body and have diverse roles, e.g. transmission in the autonomic nervous system and sensing tissue damage [52,53].

### 2.6 Transient receptor potential channels

Transient receptor potential (TRP) channels were first identified in *Drosophila*, where they have a role in photoreception in the visual system. Six TRP protein families have been identified in mammals: canonical TRP channels (TRPC) which are similar to the *Drosophila *channel, the vallanoid receptors (TRPV), melastatin TRPs (TRPM), the mucolilpins (TRPML), the polycystins (TRPP) and the ankyrin transmembrane proteins (ANKTM1 and TRPA1). All of these channels are predicted to have six TMD and all are thought to assemble as tetramers to form functional channels. All are cation selective, but most discriminate poorly between cations [54]. TRPV5 and TRPV6, however, are selective for Ca^2+ ^against monovalent cations, whereas TRPM4 and TRPM5 are selective for monovalent cations. The best characterised are the TRPV family: TRPV1 is the capsaicin receptor which is also activated by an increase in temperature, TRPV4 is thought to play a role in osmosensing, TRPV5 and TRPV6 are Ca^2+^-selective channels which have a central role in transepithelial Ca^2+ ^transport in the kidney and intestine [55]. Other well characterised channels are TRPM8 which is sensitive to menthol and cold, and TRPM6 which has a role in magnesium transport in the kidney [54].

## 3 Ion channel expression and function in the choroid plexus

### 3.1 Single channel studies

The earliest patch clamp experiments on choroid plexus examined single channel activity in tissue from the amphibian "mudpuppy" *Necturus maculosa *(see Table 1). These studies identified the expression of BK_Ca _channels with a conductance of 180 pS in the apical membrane of the epithelial cells [56,57]. Further whole-cell experiments indicated that these BK_Ca _channels carried the bulk of the whole cell conductance in amphibian tissue [58]. Christensen *et al *[59] also identified at least three anion channels with different conductances in *Necturus *choroid plexus. The importance of these channels to whole cell conductance, however, was not determined. One final important observation to emerge from these single channel experiments was the activation of non-selective cation channels by cell swelling [60]. Christensen [60] went on to show that Ca^2+ ^influx via these non-selective cation channels caused the activation of BK_Ca_. This sequence of events has since become something of a paradigm in the initiation of cell volume regulatory mechanisms in many cells.

**Table 1:** Ion channel expression in choroid plexus epithelial cells.

	**Molecular Identity**	**Functional evidence**	**References**
**K^+ ^channels**	Not determined	Ca^2+^-activated K^+ ^(Amphibia; WC;SC)	[56-58]
	Kv1.1, Kv1.3, Kv1.6	Outward rectifying conductance (WC)	[65, 69]
	Kir 7.1	Inward-rectifying current (WC)	[65, 66]
	Kir3.4	None (WC)	[68]
	TASK1	Not determined	[75]
**Anion channels**	Unknown	Inward-rectifying conductance (WC)	[79, 85]
	Unknown	Volume-sensitive conductance (WC)	[80]
**Na^+ ^channels**	ENaC	None (WC)	[90]
**Ca^2+ ^channels**	Unknown	Receptor-activated Ca^2+ ^influx (fura2)	[27, 92]
**Receptor-operated channels**	P2X	Not Determined	[94, 95]
**TRP channels**	TRPV4	Stretch-activated, non-selective cation channel in *Necturus *(SC).	[59, 98, 99]
	TRPM3	Non-selective conductance (WC)	[101, 103]
**Aquaporin-1**	AQP1	cGMP-activated, non-selective cation channel activated (WC)	[104]

WC = recorded by whole cell methods; SC = single channel recording; fura2 measurement of intracellular Ca^2+ ^activity; None = significant currents have not been observed in electrophysiological experiments; Not determined = the appropriate molecular identification or electrophysiological experiment has yet to be performed; unknown = the molecular identity of the channel protein is not known.

There are only three reports of single channel activity in mammalian choroid plexus. Somewhat surprisingly there were significant differences between the channels observed in rodent choroid plexus compared to those identified in *Necturus*. Garner and Brown [61] observed two types of anion channels in rat choroid plexus with single channel conductances of 25 and 400 pS. In a subsequent study the open probability (activity) of the 25 pS channel was shown to be increased by serotonin acting at 5HT_2C _receptors [62]. A similar effect of serotonin on anion channel activity in mouse choroid plexus had previously been reported by Hung *et al *[63], who had also observed the simultaneous inhibition of a 10 pS K^+ ^channel.

### 3.2 Whole cell patch clamp experiments on mammalian choroid plexus

Single channel experiments provided our first insight into the diverse range of ion channels expressed in the choroid plexus. However, the lack of consistency between data from mammalian and amphibian choroid plexus, coupled to the difficulty in observing any single channel activity at all in mammalian choroid plexus, prompted my laboratory to switch to the whole cell method to study the mammalian choroid plexus. These studies have yielded more consistent results, and we have identified the expression of K^+^channels, anion channels and non-selective cation channels in choroid plexus tissue from rats and mice (Table 1). Channel expression has also been studied using molecular localisation techniques, i.e. RT-PCR, northern blotting, Western blotting and immunocytochemistry (see Table 1). The remainder of this review discusses these data, and comments on the potential roles of the channels identified in the choroid plexus epithelium.

#### 3.2.1 K^+ ^channels in mammalian choroid plexus

K^+ ^channels are thought to have a number of important roles in CSF secretion. First, they help regulate the negative Vm, and hence contribute to the electrochemical gradient favouring anion efflux at the apical membrane. Second, they act as leak pathway in the apical membrane for K^+ ^accumulated in the cell through the actions of the Na^+^-K^+ ^ATPase (which is also located in the apical membrane of the choroid plexus) and thus prevent cell swelling as a result of K^+ ^accumulation. Finally, they may participate in the transcellular transport (CSF to blood) of K^+^[64]. This is an important process which is potentially vital in maintaining the low [K^+^] of the CSF, as it is thought to counteract the paracellular leak of K^+ ^from blood to CSF (i.e. ion movement through the junctional complexes between the cells). In a model for transcellular K^+ ^transport, Zeuthen and Wright [64] proposed that K^+ ^is actively pumped into cells from the CSF across the apical membrane by the Na^+^-K^+ ^ATPase. Much of this K^+ ^(about 90%) is recycled across the apical membrane through the K^+ ^channels in this membrane. However, some of the K^+ ^(the remaining 10%) will leave the cell across the basolateral membrane. Thus there is a small net absorptive (CSF to blood) flux of K^+ ^across the epithelium.

##### Kir channels

**Figure 4:**
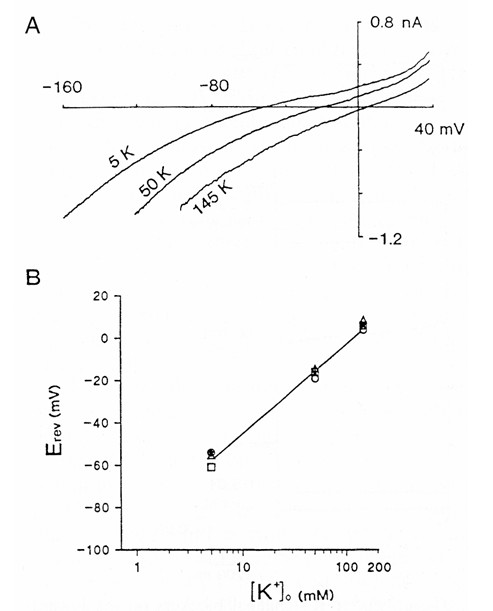
Properties of the inward-rectifying K^+ ^channel Kir7.1 expressed in rat choroid plexus epithelial cells. **A) **Current -voltage relationships with extracellular K^+ ^of 5, 50 or 145 mM. The current-voltage relationships were produced by applying 0.8 s ramp potentials from Vm = -160 to 40 mV. **B) **Kir reversal potentials (E_rev_) as a function of extracellular K^+ ^activity ([K^+^]_o_). E_rev _was measured in experiments similar to those in A (n = 3). The line through the data was predicted by the Nernst equation for a conductance which is perfectly selective for K^+^. Figure reproduced with permission from Kotera and Brown [65].

Kotera and Brown [65] characterised an inward-rectifying conductance observed in choroid plexus (Figure 4). This conductance is highly selective to K^+ ^(Figure 4B), displays no time-dependent activation or inactivation, and is blocked by sub-millimolar concentrations of Cs^+ ^or Ba^2+ ^[65]. It therefore shares many properties with conductances carried by many Kir channels. One distinguishing characteristic of the channel in the choroid plexus is that the chord conductance (i.e. the slope of the current-voltage relationship for the negative currents), is independent of the external K^+ ^concentration (see Figure 4A), whereas for most Kir channels the conductance increases with K^+^. It is now thought that the choroid plexus conductance is carried mainly by Kir7.1 channels (Table 1).

The first indication for Kir7.1 involvement came from *in situ *hybridisation studies which showed that mRNA for Kir7.1 is highly expressed in the choroid plexus epithelium [66]. Döring *et al *[66] also showed that Kir7.1, when expressed in *Xenopus *oocytes, gives rise to an inward-rectifying conductance with functional properties that are almost identical to those of the Kir in the choroid plexus, i.e. the conductance is independent of extracellular K^+ ^activity. Nakamura *et al *[67] demonstrated that the Kir7.1 channel protein is expressed in the apical membrane of the choroid plexus using immunocytochemical methods. In this membrane the Kir7.1 channel can contribute to the leak of K^+ ^from the cells and help maintain a negative Vm.

Iizuka *et al *[68] reported Kir3.4 expression in the rat choroid plexus using both immunocytochemical and *in situ *hybridisation methods. The Kir3.4 channel forms heteromeric, G protein activated K^+ ^channel with other Kir proteins (usually another member of the Kir 3 family). To date, however, electrophysiological studies have failed to identify a contribution from such channels to the whole cell conductance of choroid plexus cells. Furthermore, RT-PCR failed to identify expression of mRNA for Kir3.1 or Kir3.4 in rat choroid plexus (Speake and Brown, unpublished data).

##### Kv1 channels

**Figure 5:**
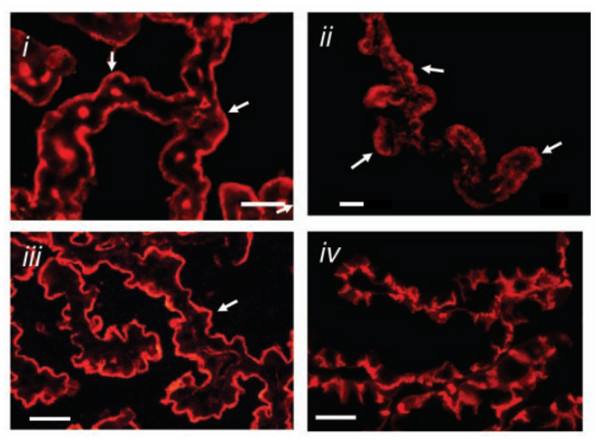
Immunoreactivity for: **i) **delayed-rectifier K^+ ^channel Kv1.3 and **ii) **Kv1.1 channel subunits in sections of rat choroid plexus tissue. The expression of **iii) **Na^+^-K^+ ^ATPase and **iv) **Cl^-^-HCO_3_^- ^exchanger AE2 are shown as markers of the apical and basolateral membranes of the epithelial cells respectively. The arrows also indicate apical staining and the scale bars = 25 μm. Figure reproduced with permission from Speake *et al *[69].

Whole cell experiments also identified outward currents in rat choroid plexus, which exhibited time-dependent activation at depolarising potentials and inactivation at extreme depolarising potentials [65]. The channels carrying this conductance were blocked by TEA^+^, dendrotoxin-K and margatoxin, the latter two toxins being highly specific blockers of Kv1.1 and Kv1.3 channels respectively [69]. Kv1 protein expression was therefore investigated by Western analysis and immunocytochemistry. Speake *et al *[69] reported that Kv1.1, Kv1.3 and Kv1.6 (but not Kv1.4 and Kv1.5) were all expressed in rat choroid plexus (Kv1.2 expression was not determined). Furthermore the immunocytochemical studies (Figure 5) demonstrated that the expression of Kv1.1 and Kv1.3 is confined to the apical membrane [69]. Kv1 channels are only expressed in a few other types of epithelial cells [70-73] where their role is not understood. A recent study, however, has demonstrated that Kv7.1 channels are important in the regulation of the Vm which drives anion secretion in intestinal epithelial cells [72]. A similar role is envisaged for the Kv1.1, Kv1.3 and possibly Kv1.6 in the choroid plexus during CSF secretion [65,69].

##### Ca^2+^-activated K^+ ^channels

BK_Ca _are expressed in amphibian choroid plexus (see Section 3.1), however, there is no evidence for the expression of these channels in mammalian tissue. Thompson-Vest *et al *[74] have demonstrated the expression of IK_Ca _in choroid plexus epithelium, expression however, appears to be confined to the cytoplasm of the cells. Indeed electrophysiological experiments have failed to identify any Ca^2+^-activated K^+ ^channels in mammalian choroid plexus cells [65].

##### Tandem pore domain K^+ ^channels

Soon after the discovery of the tandem pore domain channels, a study of the rat CNS identified expression of the acid-sensitive K^+ ^channel TASK-1 in the choroid plexus of the third ventricle using immunocytochemical methods [75]. However, it is not clear from these studies whether TASK1 is expressed in the epithelial cells of the choroid plexus or in the underlying connective and vascular tissue. Furthermore a TASK1 component to the whole-cell K^+ ^conductance has not yet been observed in patch clamp experiments (Millar & Brown, unpublished observation).

##### K^+ ^channels in the basolateral membrane

Zeuthen & Wright [64] predicted that the basolateral membrane of the choroid plexus must also express K^+ ^channels which are required to explain the net absorptive flux of K^+ ^across the epithelium (CSF to Blood). To date K^+ ^channel expression has not been observed in this membrane. A possible explanation is that in the mammalian choroid plexus K^+ ^efflux at the basolateral membrane is mediated via the K^+^-Cl^- ^cotransporter (KCC3) which is expressed at this membrane in rat choroid plexus [76]. It is possible, however, that K^+ ^channels identified by molecular methods but not yet by electrophysiology may also contribute to K^+ ^efflux at the basolateral membrane (e.g. TASK-1).

##### K^+ ^channel conclusions

Mammalian choroid plexus epithelial cells exhibit two K^+ ^conductances. One is carried by Kir7.1 channels, and one by Kv1.1 and Kv1.3 channel proteins. These channels are all expressed in the apical membrane of the choroid plexus (Figure 5) where they can mediate the reflux of K^+ ^pumped into the cells by Na^+^, K^+ ^ATPase, and help maintain the intracellular negative Vm of the epithelial cell.

#### 3.2.2 Anion channels

**Figure 6:**
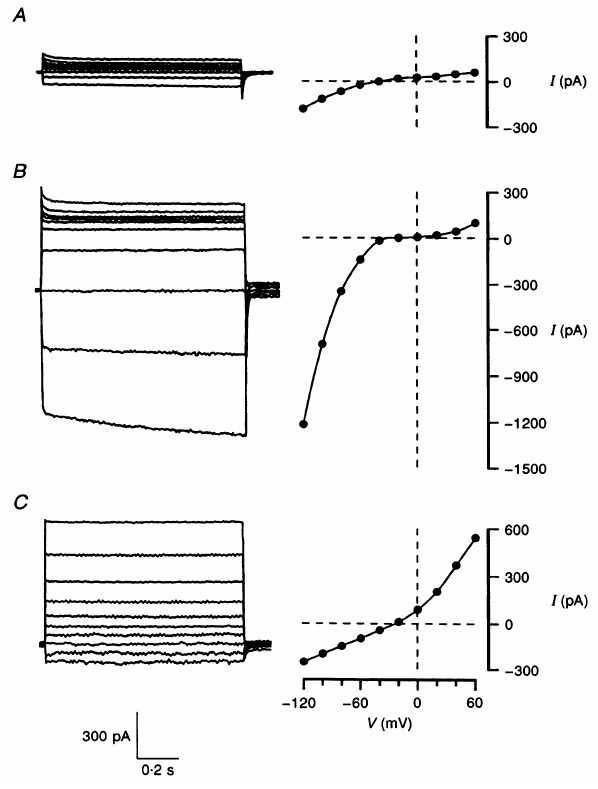
Whole cell Cl^- ^conductances in rat choroid plexus. Current profiles and current-voltage relationships for: **A) **an unstimulated cell, **B) **a cell stimulated with protein kinase A and **C) **a cell swollen by reducing the osmolality of the extracellular solution. In each case voltage clamp experiments were performed in which Vm was held at -80 mV and then stepped for 1 s to Vm from -120 to +60 mV (at 20 mV increments). Figure reproduced with permission from Kibble *et al *[79].

Anion channels are vital components of the secretory process in most epithelia, because they are normally the main route for anion efflux across the apical membrane [33]. Saito and Wright [77,78] proposed that HCO_3_^- ^permeable, apical anion channels have a major role in CSF secretion by bull frog choroid plexus. Initial patch clamp experiments on mammalian choroid plexus showed that there was very little anion conductance in unstimulated cells (Figure 6A). Subsequent experiments, however, found that two types of anion channel could be activated: i) a cAMP-activated, inward-rectifying anion channel (Figure 6B), and ii) a volume-sensitive anion channel (Figure 6C).

##### Inward-rectifying anion conductance

Anion channels with inward-rectifying current-voltage relationships have been observed in whole cell recordings from the choroid plexus of rat [79], mouse [80] and pig [81] (Table 1). These channels: i) exhibit time-dependent activation at hyperpolarising Vm (Figure 6B), ii) have a uniquely high permeability to HCO_3_^- ^(P_HCO3_:P_Cl _= 1.5), iii) are more permeant to I^- ^than Cl^- ^or Br^-^; iv) are blocked by the Cl^- ^channel blockers DIDS and NPPB, v) are activated by cAMP and protein kinase A, but inhibited by protein kinase C [79,82,83]. Many of these properties are similar to those of the ClC-2 channel [33], the mRNA for which is expressed the choroid plexus [84,85]. However, the inward-rectifying conductance was unchanged in whole cell recordings in choroid plexus cells from ClC-2 knock-out mice, indicating that ClC2 channels do not contribute to the conductance [85]. Thus, the molecular identity of the inward-rectifying channel remains unknown. The potential role of ClC-2 in the choroid plexus is also unknown, however, the data from the knock-out mice suggest that this channel is not expressed in the plasma membrane of choroid plexus cells.

The regulation of the inward-rectifying channels by cAMP and their high permeability to HCO_3_^-^, indicates that they may be similar to the HCO_3_^- ^channel thought to have a major role in CSF secretion by bullfrog choroid plexus [78]. The properties are also consistent with the observation that Cl^- ^efflux from the rat choroid plexus is stimulated by cAMP [86], and inhibited by agonists such as vasopressin which activate protein kinase C [87]. To participate in CSF secretion the inward-rectifying channels must be located in the apical membrane, but the site of their expression has yet to be determined. In this regard, recent immunocytochemical experiments have identified the expression of the electrogenic Na^+^-HCO_3_^- ^cotransporter (NBCe2) in rat choroid plexus [88]. This transporter may also have a role in HCO_3_^- ^secretion [88]. It also generates small electrical currents which will contribute to the whole cell current, thus it is likely that the P_HCO3_:P_Cl _for the inward-rectifying channel may be an overestimate of the true value.

##### Volume-sensitive anion conductance

Volume-sensitive anion channels are also expressed in choroid plexus cells from rats and mice [79,80] (Table 1). These channels are activated by cell swelling, and are dependent on intracellular ATP [80]. The channels exhibit slight outward-rectification (see Figure 6C), are blocked by DIDS and NPPB [80], and have significant HCO_3_^- ^permeability (Millar and Brown, unpublished observation). These properties are therefore similar to those of volume-sensitive anion channels found in many cells [33,46] and of the channels described by Saito & Wright [78]. Volume-sensitive channels have an important role in the regulatory volume decrease observed in most cells in response to cell swelling [33,46]. The regulation of cell volume has not been studied in the mammalian choroid plexus, but it is conceivable that these channels could be involved in both volume regulation and CSF secretion.

##### CFTR and Ca^2+^-activated Cl^- ^channels

In 1993 it was reported that CFTR was expressed in the choroid plexus on the basis of Western blotting and immunocytochemical experiments [89]. By contrast we were unable to detect mRNA for CFTR in rat choroid plexus. Furthermore, there were no differences between the anion currents in wild-type and CFTR knock-out mice [80]. Thus it is now thought that CFTR is not expressed in the choroid plexus epithelium, and that the antibody used in the immunocytochemical studies [89] may have lacked specificity for CFTR. There are no functional data to support the expression of Ca^2+^-activated Cl^- ^channels in the choroid plexus. The expression of the CLCAs and the bestrophins has not therefore been investigated.

##### Anion channel conclusions

An inward-rectifying anion conductance and a volume-sensitive anion conductance are expressed in the choroid plexus epithelium. The molecular identity of neither conductance has been determined, and the membrane in which they are expressed is also not known. Both conductances, however, could contribute to the secretion of Cl^- ^and HCO_3_^- ^into the CSF, if they are expressed in the apical membrane of the epithelium.

#### 3.2.3 Na^+ ^channels

Choroid plexus epithelial cells do not display any electrical excitability. It is therefore highly unlikely that Na_v _channels will be expressed in these cells. This conclusion is supported by data from whole cell patch clamp studies, which have failed to reveal any transient currents at depolarising potentials, which could be carried by Na_v _(or Ca_v _channels).

A recent RT-PCR and immunolocalisation study (Table 1) suggested that ENaC is expressed in the choroid plexus epithelium [90]. Amiloride which blocks ENaC certainly inhibits Na^+ ^transport into the choroid plexus [91]. This observation has always, however, been interpreted as being due to an effect of amiloride on Na^+^-H^+ ^exchangers (NHE). We have therefore performed patch clamp experiments to investigate any contribution of ENaC to the whole-cell conductance of the choroid plexus cells. These studies showed that amiloride is without effect on the whole-cell conductance of mouse cells (Millar and Brown, unpublished observations). Thus, if ENaC is expressed in choroid plexus epithelial cells, it makes only a very minor contribution to the whole cell conductance.

#### 3.2.4 Ca^2+ ^channels

There are no molecular or electrophysiological data to indicate that voltage-gated Ca^2+ ^channels are expressed in the choroid plexus epithelium. By contrast it seems likely that some form of store-operated Ca^2+ ^pathway may be expressed, e.g. Orai1 [27]. Watson *et al *[92] and Albert *et al *[93] have reported agonist-induced increases in intracellular Ca^2+ ^activity in rat and sheep choroid plexus cells respectively (Table 1). In both studies the majority of the increase was thought to be due to Ca^2+ ^release from intracellular stores, but a component may also be due to Ca^2+ ^involved entry via store-operated channels. In neither study however, was this directly tested [92,93].

#### 3.2.5 Receptor operated channels

RT-PCR experiments have determined the expression of mRNA for P2X_1_, P2X_2_, P2X_4_, P2X_5 _P2X_6 _and P2X_7 _in choroid plexus [94,95] (Table 1). Immunocytochemistry has also shown that the same P2X proteins are expressed in epithelial cells of the choroid plexus, and not in capillary endothelial cells [94,95]. The subcellular localisation of protein expression in the epithelium is not clear from data provided, and there appears to be expression on both apical and basolateral membranes [94]. There is however, some indication that expression may be greater on the apical membrane, particularly for P2X_1 _and P2X_6 _receptors[94,95]. If these data are correct then they suggest that the P2X receptors on the epithelial cells may respond to ATP in CSF, possibly as some sort of feedback loop in controlling the process of CSF secretion. Functional studies to investigate this possibility are therefore eagerly awaited.

Evidence for GABA_A _receptor expression in the choroid plexus comes from studies of benzodiazepine binding [96,97] and one of muscimol binding (a GABA_A _agonist) [98]. Furthermore, Williams *et al *[97] reported that benzodiazepines may inhibit the rate of CSF secretion. The possible expression of GABA_A _receptors in the choroid plexus, however, has not been substantiated by molecular or electrophysiological methods.

#### 3.2.6 TRP channels

**Figure 7:**
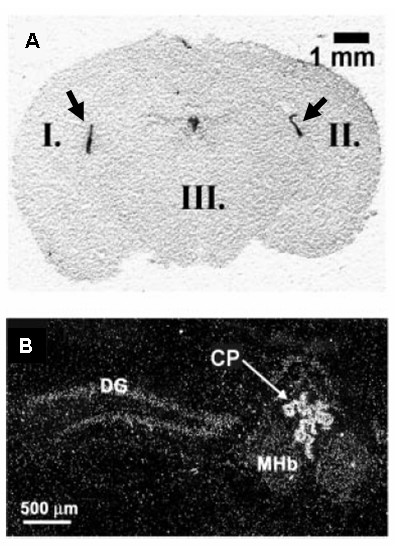
*In situ *hybridisation for the melastatin transient receptor potential channel, TRPM3 in mouse brain. mRNA for TRPM3 was detected in **A) **the choroid plexus of the lateral ventricles (see I and II) and **B) **the third ventricle (see CP). Figure reproduced with permission from Oberwinkler *et al *[101].

At least two TRP channels may be expressed in choroid plexus tissue (Table 1). TRPV4 protein has been identified in the third [99] and fourth ventricle [99,100] choroid plexus of mouse brain by immunochemistry. By contrast, i*n situ *hybridisation methods were used to demonstrate the expression mRNA encoding TRPM3 in the lateral and third ventricle choroid plexus of mouse brain (see Figure 7) [101]. The TRPV4 and TRPM3 channels are both non-selective cation channels which exhibit a finite permeability to Ca^2+ ^[102]. Both channels are activated by changes cell volume when the extracellular osmolality is perturbed, and both may have a role in cell volume regulation and/or modulating ion and water transport across epithelial barriers [102].

Patch clamp experiments in our laboratory have identified a non-selective cation conductance in mouse choroid plexus cells. The kinetic properties of the conductance mean that it is difficult to differentiate from the inward-rectifying anion conductance. Preliminary experiments however, indicate that the channel is permeable to Na^+ ^or Cs^+ ^but impermeable to the organic cation n-methyl-D-glucamine [103]. The conductance is also inhibited by 100 μM gadolinium (Gd^3+^) [103]. These data suggest that the conductance may be carried by TRPM3 or TRPV4 channels.

## 4 Unconventional ion channels

**Figure 8:**
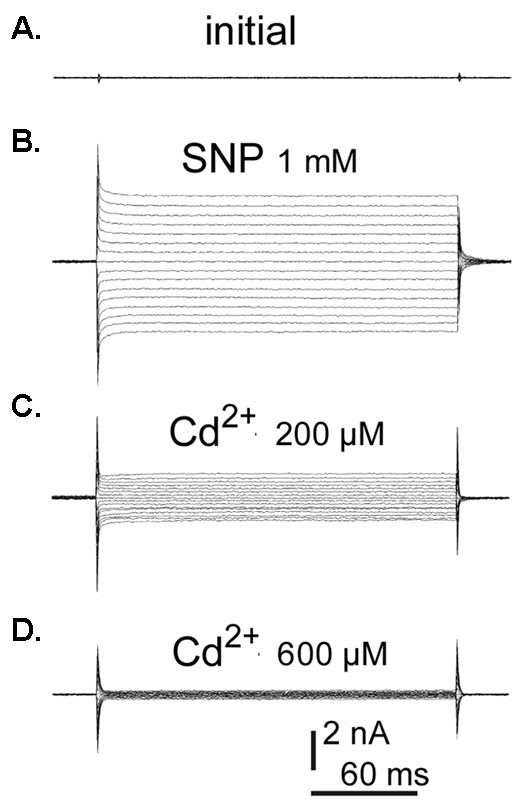
AQP1 acts a non-selective cation ion conductance which can be activated by sodium nitroprusside (SNP) activates. Currents were recorded in: **A **(**initial**) unstimulated cells; **B **(**SNP 1 mM) **in the presence of 1 mM SNP (a nitric oxide donor which activates cGMP synthesis;); **C **(**Cd^2+ ^200 μM**) in 1 mM SNP plus 200 μM Cd^2+ ^and **D **(**Cd^2+ ^600 μM**) in 1 mM SNP plus 600 μM Cd^2+^). In each experiment Vm was stepped for 200 ms from -110 to +40 mV at 10 mV increments. Figure reproduced with permission from Boassa *et al *[104].

A surprising recent finding is that aquaproin 1 (AQP1) may act as both a water channel and a ion channel in the choroid plexus [104]. AQP1 was already known to be expressed at high concentrations in the apical membrane of the mammalian choroid plexus, and is thought to play an important role in water transport by the choroid plexus [105,106]. In 2000, however, Andrea Yool and colleagues reported that AQP1 can function as a cGMP-gated, non-selective cation channel when expressed in *Xenopus *oocytes [107]. These studies were then extended to AQP1 expressed in rat choroid plexus epithelial cells [104]. It was found that a non-selective cation conductance was activated by atrial naturetic peptide (ANP), which acts to increase intracellular concentrations of cGMP [104]). Figure 8 shows the activation of the non-selective channel in choroid plexus cells by sodium nitroprusside (SNP, a nitric oxide donor which stimulates cGMP synthesis). The activated currents show no voltage-dependence (Figure 8B) and discriminate poorly between monovalent cations [104]. The AQP1 currents were inhibited by 200μM Cd^2+ ^(Figure 8C) and 600 μM Cd^2+ ^(Figure 8D). The conductance observed was thought not to be due to the activation of endogenous channels by AQP1 because: 1) the channel properties are distinct from all known endogenous channels, and 2) because the conductance could be reduced by siRNA targeted at AQP1 [104].

The biophysical relationship between the water channel and ion channel phenotype has not been established. The prevailing hypothesis is that the two different phenotypes represent differences in protein folding, with vast majority of proteins exhibiting the water channel phenotype [108]. The potential role of the AQP1 mediated conductance in the choroid plexus is also unknown. Boassa [104] did, however, observe that ANP reduced fluid secretion by choroid plexus cells, an effect which was partially reversed by 500 μM Cd^2+^. These data suggest that the activation of the non-selective conductance reduces secretion, possibly be dissipating the ion gradients across the cell membrane which are required for secretion.

## 5 Conclusion

The epithelial cells of the choroid plexus, which secrete CSF, express two K^+ ^and two anion conductances. The properties of each conductance are such that they could all play a significant role in CSF secretion. The precise role of each, however, remains to be determined. In addition there is now evidence for the expression of a number of other channel proteins, e.g. P2X receptors and TRP channels. Patch clamp experiments are required to determine the functional roles of these channels in the choroid plexus.

## Abbreviations

ABC adenosine triphosphate binding cassette

ADP adenosine diphosphate

ATP adenosine triphosphate

BK_Ca _"maxi" Ca^2+^-activated K^+ ^channels

Ca_v _voltage-gated Ca^2+ ^channels

CFTR cystic fibrosis transmembrane conductance regulator

ClC a family of voltage-dependent channels and transporters

CNS central nervous system

DIDS 4-4' diisothiocyanatostilbene-2,2'-dislphonic acid (an anion channel blocker)

ENaC epithelial Na^+ ^channels

fS 	femto (10^-15^) siemen (a measure of electrical conductance)

IK_Ca _intermediate conductance Ca^2+^-activated K^+ ^channels

Kir inward-rectifying K^+ ^channel

Kv delayed-rectifier K^+ ^channel

Na_v _voltage-gated Na^+ ^channel

NPPB 5-nitro-2-(3-phenylpropylamino)benzoic acid (an anion channel blocker)

P_HCO3_:P_Cl _channel permeability to HCO_3_^- ^relative to Cl^- ^permeability

P domain pore forming domain of an ion channel

pS pico (10^-12^) siemen

ROC receptor operated channel

RT-PCR reverse transcriptase polymerase chain reaction

S transmembrane segment

siRNA interference RNA knock down

SK_Ca _small conductance Ca^2+^-activated K^+ ^channels

SOCE store-operated Ca^2+ ^entry

TMD transmembrane domain

TRP transient receptor potential

UDP uridine diphosphate

UTP uridine triphosphate

Vm membrane potential

This list only includes frequently used standard abbreviations. Many channel proteins have abbreviated which are used as the accepted name, these are defined where they arise in the text.

## Competing interests

The author(s) declare that they have no competing interests.

## Authors' contributions

IDM – Joint principal author

JIEB – Author of Section 2.3.2 and reading the manuscript

PDB – Joint principal author

All authors have read and approved the final version of the manuscript.
